# Mondor's Disease Following Augmentation Mammoplasty: A Case Report

**DOI:** 10.7759/cureus.117059

**Published:** 2026-09-28

**Authors:** João Castro e Silva, Marta Ferreira, Inês Pereira, Sara Anjos

**Affiliations:** 1 Unidade de Saúde Familiar Ponte, Unidade Local de Saude do Alto Ave, Guimarães, PRT

**Keywords:** mammaplasty, mondor disease, postoperative complications, surgery, thrombophlebitis

## Abstract

Mondor's disease is a benign, self-limiting superficial thrombophlebitis affecting the thoracic or abdominal wall, which can occur following local trauma or surgical manipulation, including breast surgery. We report the case of a previously healthy 42-year-old woman who underwent bilateral augmentation mammoplasty for aesthetic purposes. Fifteen days after surgery, she developed discomfort and a sensation of tight subcutaneous cords, initially in the abdominal region and subsequently in the inframammary area. Approximately one month after surgery, physical examination revealed palpable, mildly tender subcutaneous cords in the inframammary region, with mild associated erythema and no signs of infection, hematoma, or implant-related complications. A clinical diagnosis of Mondor's disease was established, and conservative management with analgesia and clinical surveillance was adopted, with spontaneous resolution within four weeks. This case highlights the importance of considering Mondor's disease in the differential diagnosis of linear cutaneous changes or palpable subcutaneous cords following breast surgery, allowing for an accurate clinical diagnosis and an appropriate conservative approach, while avoiding unnecessary investigations and interventions.

## Introduction

Mondor's disease is a rare, generally benign, and self-limiting superficial thrombophlebitis affecting the subcutaneous veins of the thoracic or abdominal wall, first described by Henri Mondor in 1939 [1]. It typically presents as a tender, cord-like induration that follows the course of the affected vein, most commonly the thoracoepigastric, lateral thoracic, or superior epigastric veins, and later evolves into a painless, fibrous band as the acute inflammatory phase subsides [2]. The condition occurs more frequently in women, generally between the second and fifth decades of life, and most commonly affects the thoracoepigastric vein [2]. It typically resolves spontaneously within four to eight weeks, without the need for specific treatment [3].

The pathophysiology of Mondor's disease remains incompletely understood [3]. It may arise idiopathically or secondary to local trauma, surgical manipulation, or an underlying hypercoagulable state or malignancy and has been described following reduction and augmentation mammoplasty [3,4]. Reported incidences range from approximately 1% following breast augmentation via an inframammary incision [5] to up to 12% among patients with breast cancer [4]. Although the association with malignancy is uncommon, Mondor's disease has been reported in temporal relation to underlying breast cancer, and routine mammography has been advised in idiopathic presentations to exclude this possibility [4,6].

In the postoperative setting, this condition may be underrecognized, as reports specifically implicating aesthetic breast surgery remain uncommon in the literature [2]. Early recognition of its typical clinical presentation is therefore important to facilitate an accurate diagnosis and, in the absence of signs suggestive of an alternative complication, support a conservative management approach without recourse to unnecessary investigations [4]. The authors report a case of Mondor's disease diagnosed during follow-up after augmentation mammoplasty, highlighting its clinical features and favorable outcome with conservative management.

## Case presentation

A previously healthy 42-year-old woman with no relevant personal history underwent bilateral augmentation mammoplasty for aesthetic purposes. The augmentation was performed through a periareolar approach, with round, microtextured, moderate projection implants of 195 mL placed in the subglandular plane bilaterally, and the immediate postoperative course was uncomplicated. Postoperatively, the patient wore a surgical bra and an intermediate compression dressing and began manual lymphatic drainage on the first postoperative day, continuing two to three times per week during the first month. On postoperative day 15, she developed discomfort and a sensation of "tight cords" beneath the skin, initially in the abdominal region and subsequently in the inframammary area.

Approximately one month after the procedure, during a regular follow-up consultation, physical examination revealed palpable, mobile, linear subcutaneous cords in the inframammary region, mildly uncomfortable on palpation, with mild associated erythema and no fluctuance (Figure 1). The patient was afebrile, with no systemic symptoms, and no palpable axillary lymphadenopathy. There were no clinical signs of infection, hematoma, or implant-related complications. Given the characteristic examination findings, the clear temporal association with recent surgery, and the absence of clinical features suggesting infection, implant complications, deep venous thrombosis, or breast pathology, a clinical diagnosis of Mondor's disease was established, and additional imaging was not considered necessary. A conservative approach was adopted, consisting of analgesia and clinical surveillance. The clinical course was favorable, with spontaneous resolution of the findings within four weeks.

**Figure 1:**
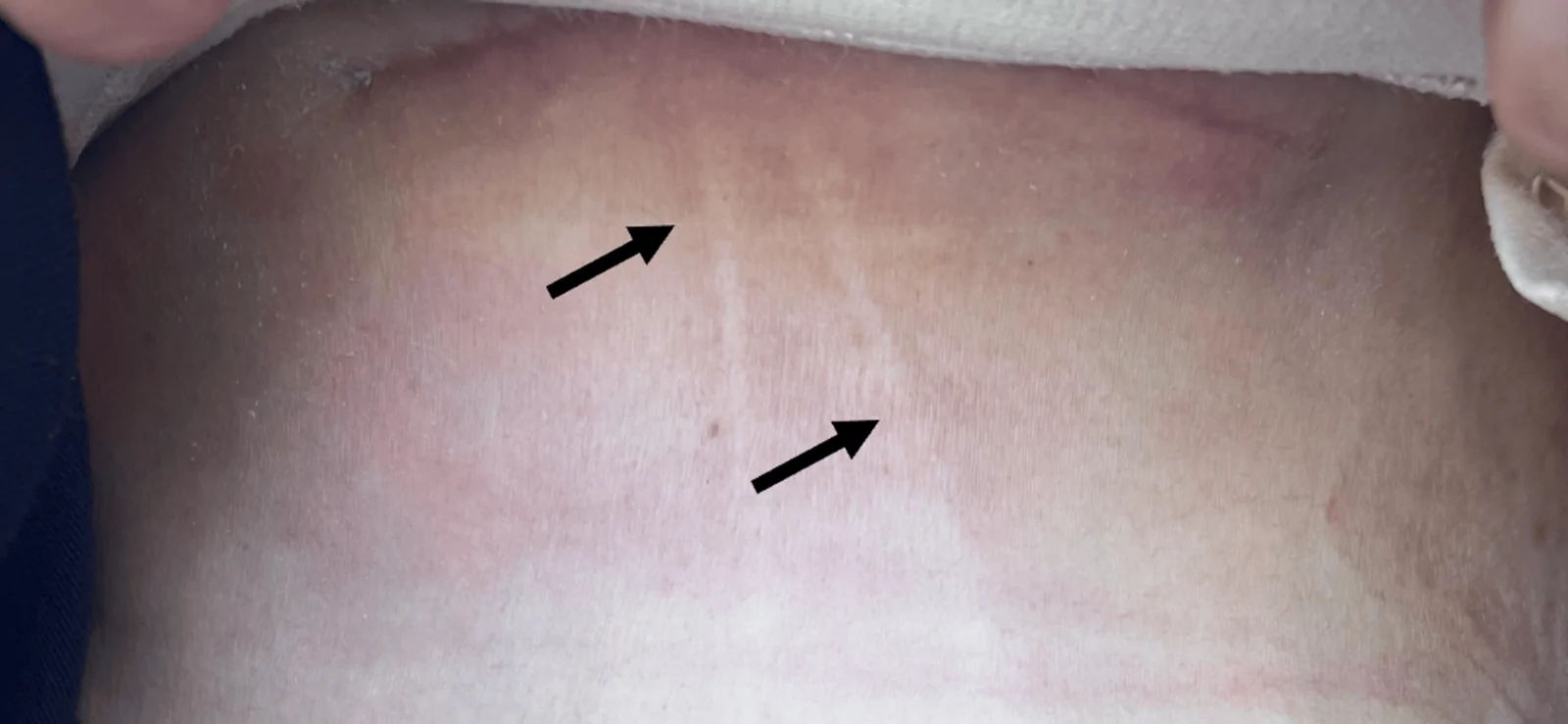
Mondor's cords under the right breast (arrows).

## Discussion

Mondor's disease following augmentation mammoplasty is a rare occurrence, with only a limited number of cases specifically reported in the aesthetic breast surgery literature [2,4,7,8]. In one retrospective series of 652 women undergoing aesthetic breast surgery over a decade, only three cases of Mondor's disease were identified, corresponding to an incidence of 0.46% [9]. Its rarity, combined with a clinical presentation that can be mistaken for more concerning postoperative complications, makes awareness of this entity particularly relevant whenever pain or linear cutaneous changes arise following breast surgery. In this case, the temporal relationship with recent surgical manipulation and postoperative compressive garments may have contributed to the venous injury underlying the clinical findings, consistent with mechanisms of trauma, transection, and external compression described in the literature [4,7], although a direct causal relationship cannot be established from a single case.

The timing of symptom onset in relation to surgery varies considerably across published cases. While some series report onset never occurring within the first two postoperative weeks [9], other reports describe presentation as early as 7 to 14 days after subfascial augmentation [10], and cases have even been documented up to 13 years after the original procedure [11], suggesting that a remote surgical history should not preclude the diagnosis. In this case, symptom onset on postoperative day 15 falls close to this early boundary, reinforcing that Mondor's disease should remain part of the differential diagnosis even in the immediate postoperative period.

The clinical hallmark of the condition is a palpable, cord-like subcutaneous induration [1,3]. Although characteristic, this presentation may raise concern for other postoperative complications following breast augmentation; however, no correlation has been demonstrated between Mondor's disease and silicone implants or capsular contracture, supporting its distinction as an independent, implant-unrelated entity [4,8]. The initial perception of cord-like changes in the abdominal wall, followed by involvement of the inframammary region, may reflect the anatomical continuity of the superficial thoracoepigastric venous network, which connects the epigastric venous system to the lateral thoracic and inframammary area. Recognition of this anatomical continuity may help clinicians identify Mondor's disease when postoperative cords extend beyond the immediate breast region [12].

Diagnosis remains predominantly clinical, based on a thorough history and physical examination [3]. When confirmation is required, ultrasonography is the modality of choice, typically demonstrating a noncompressible, hypoechoic tubular structure without flow on color Doppler imaging, occasionally corresponding to a linear density on mammography [13]. Imaging and further investigation are particularly recommended when the clinical presentation is atypical, when diagnostic uncertainty persists, or in the absence of a clear precipitating event such as recent trauma or surgery, given the reported association with underlying breast malignancy [4,6]. Similar diagnostic vigilance has been advocated even when Mondor's disease develops as a complication of other breast procedures, such as core needle biopsy, reinforcing that its recognition should not distract from the need to exclude other pathology when the presentation is not entirely straightforward [14].

Management is conservative in the majority of cases, with resolution typically observed within weeks. Reported approaches range from reassurance and simple analgesia to nonsteroidal anti-inflammatory drugs and, occasionally, antithrombotic therapy in select cases with more extensive or persistent thrombosis, although no single strategy has been shown to accelerate resolution [4,14]. This conservative approach is consistent with the favorable course observed in the present patient.

In this case, symptom onset coincided temporally with postoperative physiotherapy sessions, although a causal relationship cannot be firmly established. This case adds to the sparse existing literature on Mondor's disease as a rare complication of augmentation mammoplasty [2,4,7-11] and reinforces that awareness of its benign, self-limiting nature is essential to guide an appropriately conservative management approach, avoiding unnecessary interventions while safely excluding more serious postoperative complications.

## Conclusions

This case underscores the importance of including Mondor's disease in the differential diagnosis of postoperative subcutaneous cords or linear skin changes following breast surgery, including augmentation mammoplasty. In typical presentations, characterized by a clear temporal association with recent surgery and the absence of alarming features, diagnosis remains predominantly clinical, without the need for additional imaging or laboratory investigation; atypical presentations or diagnostic uncertainty, however, may still warrant further investigation. Recognition of this benign, self-limiting entity supports a conservative management approach, sparing patients unnecessary interventions while providing appropriate reassurance, as illustrated by the favorable outcome observed in this case.
